# Probing the Growth Improvement of Large-Size High Quality Monolayer MoS_2_ by APCVD

**DOI:** 10.3390/nano9030433

**Published:** 2019-03-14

**Authors:** Tao Han, Hongxia Liu, Shulong Wang, Shupeng Chen, Wei Li, Xiaoli Yang

**Affiliations:** 1 Key Laboratory for Wide-Band Gap Semiconductor Materials and Devices of Education, the School of Microelectronics, Xidian University, Xi’an 710071, China; 15639119745@163.com (T.H.); chenshupeng999@126.com (S.C.); li20101467@163.com (W.L.); 2 The School of Mathematics and Statistics, Xidian University, Xi’an 710071, China; xiaoyangxiaoli163@163.com

**Keywords:** monolayer MoS_2_, APCVD, Raman spectra, growth condition, PL spectrum, graphene quantum dot, Oxygen plasma

## Abstract

Two-dimensional transition metal dichalcogenides (TMDs) have attracted attention from researchers in recent years. Monolayer molybdenum disulfide (MoS_2_) is the direct band gap two-dimensional crystal with excellent physical and electrical properties. Monolayer MoS_2_ can effectively compensate for the lack of band gap of graphene in the field of nano-electronic devices, which is widely used in catalysis, transistors, optoelectronic devices, and integrated circuits. Therefore, it is critical to obtain high-quality, large size monolayer MoS_2_. The large-area uniform high-quality monolayer MoS_2_ is successfully grown on an SiO_2_/Si substrate with oxygen plasma treatment and graphene quantum dot solution by atmospheric pressure chemical vapor deposition (APCVD) in this paper. In addition, the effects of substrate processing conditions, such as oxygen plasma treatment time, power, and dosage of graphene quantum dot solution on growth quality and the area of the monolayer of MoS_2_, are studied systematically, which would contribute to the preparation of large-area high-quality monolayer MoS_2_. Analysis and characterization of monolayer MoS_2_ are carried out by Optical Microscopy, AFM, XPS, Raman, and Photoluminescence Spectroscopy. The results show that monolayer MoS_2_ is a large-area, uniform, and triangular with a side length of 200 μm, and it is very effective to treat the SiO_2_/Si substrate by oxygen plasma and graphene quantum dot solution, which would help the fabrication of optoelectronic devices.

## 1. Introduction

Molybdenum disulfide (MoS_2_) has attracted attention from researchers in recent years because of its excellent physical properties. MoS_2_ has been studied extensively in the field of two-dimensional nano-electronic devices, which has the potential to become an alternative to grapheme [1,2,3,4]. MoS_2_ is a two-dimensional transition metal dichalcogenides (TMDs) with an adjustable band gap structure in which atoms in layers are bonded by covalent bond interaction, whereas interlayer atoms are coupled by weak van der Waals forces [5,6]. Monolayer MoS_2_ has excellent photoelectric properties due to its high electron mobility and unique band gap structure. The bulk MoS_2_ is an indirect bandgap semiconductor with band gap energy about 1.29 eV, band gap energy increases with the decrease of MoS_2_ layers number, thus monolayer MoS_2_ becomes the direct bandgap semiconductor with 1.90 eV [7,8,9]. Due to the existence of a direct band gap, field effect transistors based on monolayer MoS_2_ have higher current switching ratio and electron mobility at room temperature [10,11]. In addition, it can also be used in phototransistors [12], logic circuits [13], secondary batteries, and photo catalysis [14].

At present, most reports on the preparation of monolayer MoS_2_ are by mechanical peeling [15], lithium ion intercalation [16,17,18], hydrothermal [19], and chemical vapor deposition (CVD) [3,4]. The above four methods are simple in operation, wide in range, but require high preparation of the environment, high cost, and have poor repeatability [20,21,22]. Moreover, the layer number of MoS_2_ is difficult to control, and the maximum area of monolayer MoS_2_ is 100 μm approximately according to existing reports, which is a disadvantage to the fabrication of optoelectronic devices, as shown in Table 1. Although the growth method of MoS_2_ is already defined, the preparation of large-area uniform monolayer MoS_2_ is still a difficult problem to be solved.

It can be seen from the above reported literature that the maximum side length of monolayer MoS_2_ is 100 μm approximately [23,24]. Effects of the substrate processing conditions, such as oxygen plasma treatment time, power, and amount of graphene quantum dot solution on growth quality and area of MoS_2_ are researched systematically in this paper. The large-area uniform triangle monolayer MoS_2_ with a side length of 200 μm is prepared on an SiO_2_/Si substrate by atmospheric pressure chemical vapor deposition (APCVD) without vacuum treatment, which can provide materials for the photovoltaic devices effectively [25]. First, this paper introduces the growth experiment of MoS_2_ and testing equipment and then introduces the influence of different SiO_2_/Si substrate treatment factors on growth morphology and quality of MoS_2_. This is followed by the characterization of monolayer MoS_2_ under optimal treatment conditions of SiO_2_/Si substrate, which was carried out by Optical Microscopy, AFM, XPS, Raman, and Photoluminescence Spectroscopy. Finally, it is concluded that it is possible to obtain the large-area high-quality monolayer MoS_2_ by treating SiO_2_/Si substrate with oxygen plasma and graphene quantum dot solution, which would contribute to the preparation of photovoltaic device.

## 2. Methods

**Figure 1:**
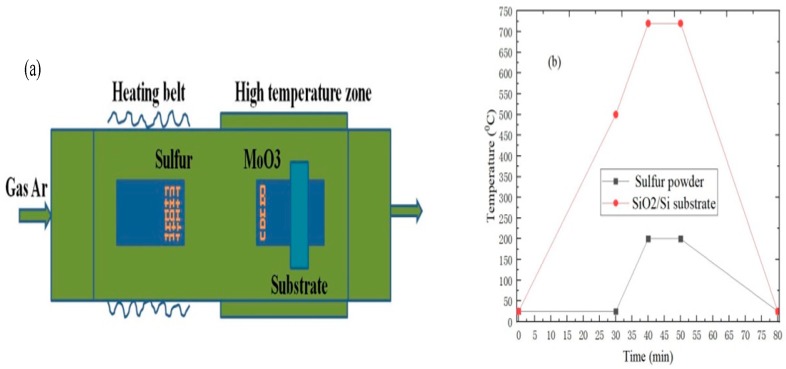
(**a**) Growth experiment schematic diagram of MoS_2_; (**b**) temperature change diagram of MoS_2_ grown by atmospheric pressure chemical vapor deposition (APCVD).

Before the MoS_2_ growth experiment, SiO_2_/Si substrates were respectively placed in absolute ethanol, acetone, and deionized water solution for ultrasonic cleaning in 10 min, 10 min, 10 min, and dried with a nitrogen gun. Compared to the reported literature, the difference is that SiO_2_/Si substrates are cleaned with PCE-6 plasma cleaner under different time and power conditions. Subsequently, different dosages of 1 mg/mL graphene quantum dot solution were applied to SiO_2_/Si substrates uniformly with oxygen plasma by using VTC-100 vacuum rotary coater. In addition, a quartz boat with 100 mg sulfur powder (Alfa Aesar, Shanghai, China, 99.5%) was transferred to the low temperature zone of the tube furnace constructed by the winding heating belt. Moreover, 2 mg MoO_3_ powder (Alfa Aesar, Shanghai, China, 99.95%) was weighed into one end of the quartz boat, and SiO_2_/Si substrate was placed faced down 5 cm apart from the MoO_3_ powder, and the quartz boat with MoO_3_ powder and SiO_2_/Si substrate was placed in the high temperature zone of tube furnace, as shown in Figure 1a. Figure 1b shows the temperature change diagram of MoS_2_ grown by APCVD, high-purity argon gas with a flow rate of 300 sccm was introduced into the tube furnace for 10 min to exclude air in the tube furnace before the start of the reaction. The high temperature zone of the tube furnace was heated from room temperature to 550 °C in 30 min, meanwhile the heating belt began to heat, and the temperature was controlled at 200 °C. The temperature of the high temperature zone continued to 720 °C in 10 min with heating, and maintained the growth temperature for 10 min before cooling down to room temperature. The temperature distribution in the furnace was as follows: The closer the distance from the intermediate heating zone of the tube furnace was, the higher the temperature was [26,27]. Finally, argon gas with a flow rate of 50 sccm was introduced into the tube furnace throughout the reaction experiment, and the pressure of the tube furnace was maintained at a normal pressure.

The new generation high-resolution Raman spectroscopy (LabRAM HR Evolution, HORIBA Jobin Yvon, Paris, France) was used to characterize the surface topography, Raman, and Photoluminescence spectrum of MoS_2_ systematically. The specific test conditions of the Raman spectroscopy were as follows: 532 nm laser with 1 μm spot diameter, spectral resolution ≤0.65 cm^−1^, scan time 5 s, and accumulations number 3 s. The layer number of MoS_2_ could be determined quickly by the difference in the contrast between MoS_2_ and SiO_2_/Si substrate under the optical microscope. Raman spectrum was the scattering spectrum that utilized the inelastic scattering effect of the molecules on photons, which can provide an efficient representation of molecular structure. Photoluminescence spectrum is a form of cold luminescence, it would re-emit photons or electromagnetic waves when SiO_2_/Si substance absorbs photons or electromagnetic waves of a certain frequency. In addition, surface morphology and thickness of MoS_2_ grown on SiO_2_/Si substrate were characterized through interatomic interaction forces by using atomic force microscopy with longitudinal high resolution (AFM, Dimension 3100, Veeco Instruments, Shanghai, China). Moreover, X-ray photoelectron spectroscopy-Auger electron spectroscopy (Theta 300 XPS system, Thermo Fisher, Shanghai, China) was also used to measure chemical and electronic states of MoS_2_.

Optical microscopy (OM) was the most efficient and intuitive method to characterize layered structural materials. MoS_2_ was grown on silicon substrate with a layer of 285 nm SiO_2_ by APCVD. The number, size and film formation of MoS_2_ can be quickly judged by the contrast between MoS_2_ and SiO_2_/Si substrate under the optical microscope.

## 3. Results and Discussion

### 3.1. Effect of Different Oxygen Plasma Treatment Time and Power on the Growth of MoS_2_

SiO_2_/Si substrates are cleaned by the PCE-6 oxygen plasma cleaning machine in this experiment. The specific cleaning steps are as follows: First put SiO_2_/Si substrate into the vacuum chamber and vacuum it to below 1 Pa. Then oxygen gas is introduced to maintain oxygen vacuum in the vacuum chamber to 50 Pa. As the substrate treatment effect is greatly affected by gas pressure, this affects discharge power and processing time during glow discharge. Subsequently, the photo-discharge is performed at the discharge frequency of 13.56 MHz, and SiO_2_/Si substrates are processed sequentially at 300 W, 500 W, and 1000 W discharge power for 30, 60, 90, and 120 s. The principle of oxygen plasma cleaning is to convert oxygen into its plasma state by the glow discharge. These high-energy ions bombard the surface of the SiO_2_/Si substrate and easily react with the surface of substrate to increase the surface energy, thereby changing the surface properties of the SiO_2_/Si substrate and remove the organic impurities of the SiO_2_/Si substrate surface, which can achieve a hydrophilic effect, and improve the surface activity of the silicon wafer [28]. The characterization of the SiO_2_/Si substrate before and after oxygen plasma cleaning treatment can be observed by the growth of MoS_2_. The specific growth of MoS_2_ under different oxygen plasma treatment time and power are as follows.

**Figure 2:**
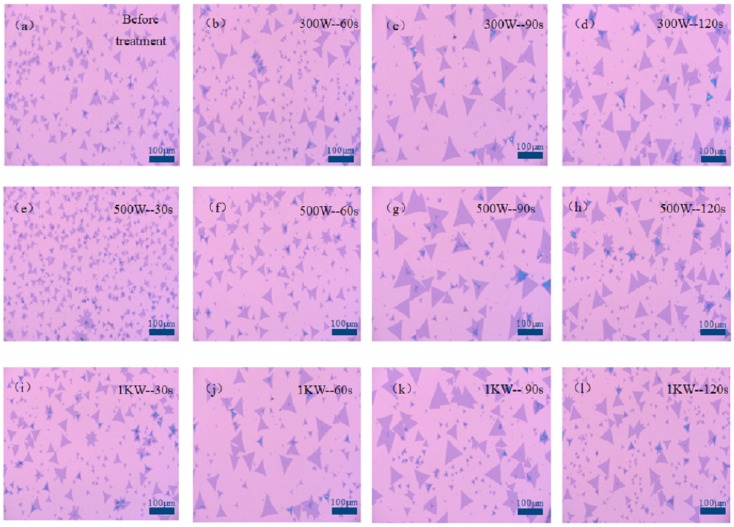
The growth of MoS_2_ on SiO_2_/Si substrate under the different oxygen plasma treatment time and power. (**a**) Before treatment, (**b**) 300 W, 60 s, (**c**) 300 W, 90 s, (**d**) 300 W, 120 s, (**e**) 500 W, 30 s, (**f**) 500 W, 60 s, (**g**) 500 W, 90 s, (**h**) 500 W, 120 s, (**i**) 1 KW, 30 s, (**j**) 1 KW, 60 s, (**k**) 1 KW, 90 s, (**l**) 1 KW, 120 s.

It can be found by observing Figure 2 that the growth and morphology of MoS_2_ would, respectively, become uniform and better, and the size was also improved with an increase of processing time when the oxygen plasma was used in the low-power and medium-power oxygen plasma cleaning of SiO_2_/Si substrate. This is because the surface roughness is improved significantly after oxygen plasma cleaning, and it can also change the surface characteristics of the SiO_2_/Si substrate. The growth of MoS_2_ initially becomes better and more uniform with the increase of processing time when the SiO_2_/Si substrate is subjected to high-power oxygen plasma cleaning, but the treatment time has almost no effect on the growth of MoS_2_ after 60 s, which indicates that too long processing time does not always increase the surface activity of the MoS_2_ material. As the oxygen plasma cleaning power increases, the density and energy of the plasma increase, and the oxygen plasma treatment speed also increases. At this time, the morphology of MoS_2_ is the triangle with uniform size, and multilayer or bulk materials of MoS_2_ also appear when the time of cleaning SiO_2_/Si substrate is constant. As shown in Figure 2, it is found that the optimal oxygen plasma cleaning power and time of SiO_2_/Si substrate processing are 500 W and 90 s, respectively. At this time, the morphology of MoS_2_ is the best, and the maximum triangular side length can be up to 100 μm.

### 3.2. Effect of Graphene Quantum Dot Solution Amount on the Growth of MoS_2_

Impurities and defects on the SiO_2_/Si substrate are easily selected when MoS_2_ sample is deposited by CVD. Therefore, the processing of SiO_2_/Si substrate would have a greater impact on the nucleation process of MoS_2_. VTC-100 vacuum rotary coating machine is used to spin the graphene quantum dot solution with a particle size of 2 nm on cleaned SiO_2_/Si substrate uniformly in the experiment, and research the effect of different dosages of 1mg/ml graphene quantum dot solution on the growth of MoS_2_ by non-uniform nucleation principle. The size, quality, and continuity of monolayer MoS_2_ are improved by dropping the graphene quantum dot solution on SiO_2_/Si substrate.

**Figure 3:**
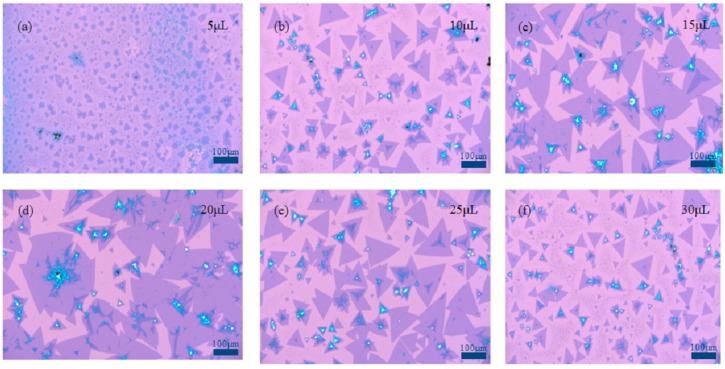
Effect of grapheme quantum dot solution with different dosage on the growth of MoS_2._ (**a**) 5μL, (**b**) 10μL, (**c**) 15μL, (**d**) 20μL, (**e**) 25μL, (**f**) 30μL.

In order to obtain the optimum dosage of graphene quantum dot solution, SiO_2_/Si substrates with 5 μL, 10 μL, 15 μL, 20 μL, 25 μL, and 30 μL of 1 mg/mL graphene quantum dot solution are carried out on the growth experiments of MoS_2_. It can be seen from Figure 3 that different dosages of graphene quantum dot solution on SiO_2_/Si substrates have an effect on growth of MoS_2_. It can be observed from Figure 3a–d that many small triangles with a size of 20 μm would appear on SiO_2_/Si substrate under the same growth conditions, the size of the triangular MoS_2_ increases gradually with graphene quantum dot solution dosage increase, large area continuous film with 300 μm lateral dimension is formed, and the middle of the MoS_2_ film is obviously thicker. This is because a certain dosage graphene quantum dot solution can provide a suitable nucleation point for MoS_2_ on SiO_2_/Si substrate, which would facilitate the growth of a large-area MoS_2_ film. In Figure 3e,f, MoS_2_ sample deposited on SiO_2_/Si substrate is the triangle with a size of 40 μm, and no uniform continuous film is formed. The reason is that the graphene quantum dots solution dosage is too high and the nucleation sites are excessive. In conclusion, the optimal dosage of 1 mg/mL graphene quantum dot solution is 20 μL by analyzing the above results.

### 3.3. The Optimal Growth Conditions of Monolayer MoS_2_

**Figure 4:**
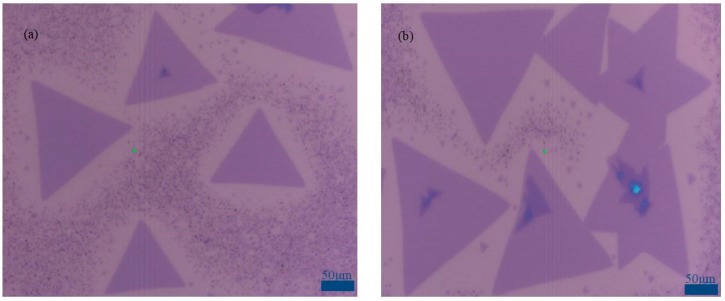
Growth of different regions MoS_2_ on SiO_2_/Si substrate under optimal treatment condition (**a**) first region; (**b**) second region Scale bar: 50 μm.

In Figure 4, the size of large-area high-quality monolayer MoS_2_ grown on SiO_2_/Si substrate with oxygen plasma (500 w, 90 s) and graphene quantum dot solution (20 μL, 1 mg/mL) can be up to 200 μm, and different monolayer MoS_2_ spliced together to form large area continuous film with uniform color and good film formation quality. The reason is that a certain dosage graphene quantum dot solution can provide a suitable nucleation point for MoS_2_, which would contribute to the growth of large-area monolayer MoS_2_ film. Monolayer MoS_2_ are characterized by Optical Microscopy, AFM, XPS, Raman, and Photoluminescence Spectroscopy. The following are the test results in detail.

### 3.4. Characterization of Monolayer MoS_2_

**Figure 5:**
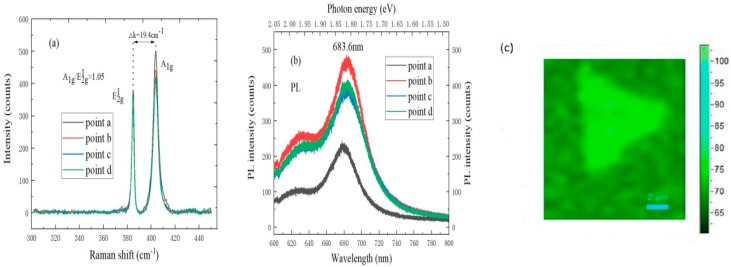
(**a**) Raman spectrum, (**b**) PL spectrum, and (**c**) mapping of monolayer MoS_2_ under optimal treatment conditions of SiO_2_/Si substrate.

It can be found by observing Figure 5a that there are two characteristic peaks in Raman spectrum of monolayer MoS_2_. The E^1^_2g_ peak position of in-plane vibration mode is located at 383.3 cm^−1^, and the A_1g_ peak position of out-of-plane vibration mode is located at 402.7 cm^−1^, the peak distance between two peaks is 19.4 cm^−1^, and A_1g_/E^1^_2g_ ≈ 1.05, which indicates the triangular MoS_2_ is monolayer [29]. In Figure 5b, monolayer MoS_2_ film has the strongest luminescence peak at 683.6 nm due to the direct excitation of excitons. It can be known from the conversion relationship between wavelength and electron volts that the corresponding transition energy level at the luminescence peak is about 1.82 eV, and there is also a B peak at 1.98 eV due to the interaction of electrons in the 3d orbital of molybdenum atoms, which can further prove that the sample is a large-area monolayer MoS_2_ with good film quality [30,31]. In addition, Raman mapping is carried out to observe film-forming quality and uniformity of triangular monolayer MoS_2_, and the color is very uniform, which also indicates that the sample is monolayer MoS_2_ with highly uniform quality.

**Figure 6:**
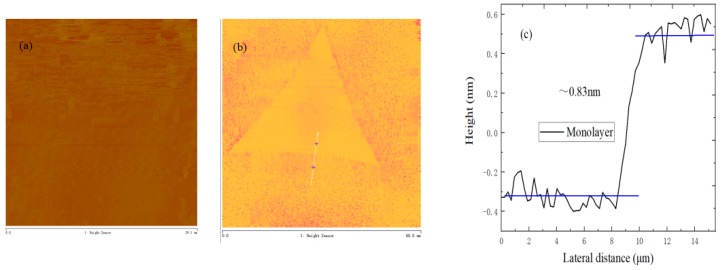
(**a**) Atomic force microscopy (AFM) topography of MoS_2_ film; (**b**) AFM topography of triangular MoS_2_; (**c**) AFM height profile of triangular MoS_2_ (blue lines represent the average height of triangular MoS_2_ under different position).

Atomic force microscopy (AFM) is used to characterize the surface topography of layered structural materials. The thickness of MoS_2_ on SiO_2_/Si substrate is characterized by AFM, as shown in Figure 6. The film and triangular MoS_2_ are very uniform in color, and the thickness of MoS_2_ is about 0.83 nm, which indicates that MoS_2_ is monolayer.

**Figure 7:**
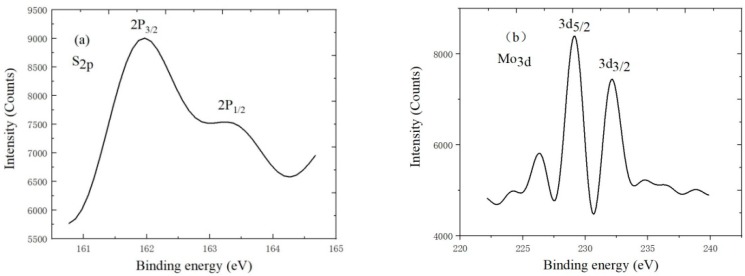
(**a**) S_2p_; (**b**) Mo_3d_ of X-ray photoelectron spectroscopy.

X-ray photoelectron spectroscopy (XPS) is the advanced analytical technique in microscopic analysis of electronic materials and components. It uses photoelectron binding energy as abscissa and relative intensity as ordinate to make photoelectron spectroscopy. The relevant information of MoS_2_ is obtained by analyzing the energy spectrum. It can be seen from Figure 7 that Mo3d_5/2_ and 3d_3/2_ binding energy in Mo_4+_ is about 228.5 eV and 232.5 eV, respectively. At the same time, 2P_3/2_ and 2P_1/2_ binding energy in S_2p_ is about 162.2 eV and 163.3 eV, respectively. The percentages of the S_2p_ element and Mo_3d_ element are 15.79% and 7.58%, respectively, which also indicates MoS_2_ is monolayer.

## 4. Conclusions

In this paper, large-area high-quality uniform triangle monolayer MoS_2_ is successfully grown on an SiO_2_/Si substrate with oxygen plasma treatment and graphene quantum dot solution by APCVD. Effects of substrate processing conditions, such as oxygen plasma treatment time, power, and the amount of graphene quantum dot solution on growth quality and the area of monolayer MoS_2_ are studied in detail. The optimal dosage of 1mg/ml graphene quantum dot solution is 20 μL, and the optimal power and time of SiO_2_/Si substrate processing are 500 W and 90 s, respectively. Analysis and characterization of monolayer MoS_2_ are carried out by Optical Microscopy, AFM, XPS Raman, and Photoluminescence Spectroscopy. The results show that the grown sample on the SiO_2_/Si substrate under optimal cleaning conditions is a large-area high-quality, uniform, triangular monolayer MoS_2_ with a side length of 200 μm, and it is very effective to treat SiO_2_/Si substrate by oxygen plasma and graphene quantum dot solution, which can pave the way for the fabrication of optoelectronic devices.

## Figures and Tables

**Table 1:** Comparison of different growth modes of MoS_2_.

Growth Method	Characteristics	Size of MoS_2_	References
Micromechanical stripping	Simple process, low yield, poor repeatability	10 µm	[15,16]
Lithium ion intercalation	Complicated operation and high cost	20 µm	[16,17,18]
Hydrothermal	Poor crystallization quality	20~30 µm	[19,20,21]
CVD	Layer number cannot control	30~50 µm	[2,3,4,8,9,10]
APCVD	Simple operation, no vacuum treatment	80~100 µm	[25]
APCVD	Oxygen plasma treatment, graphene quantum dot	200 µm	This paper
